# Predictors of Smartphone Usage Addiction among Health Sciences Students in Selected Universities in Kampala, Uganda

**DOI:** 10.24248/eahrj.v8i3.811

**Published:** 2025-01-30

**Authors:** Abdulmujeeb Babatunde Aremu, Ismail Bamidele Afolabi, Naziru Rashid

**Affiliations:** a Faculty of Health Sciences, Islamic University in Uganda, Kampala Campus, Kampala Uganda; b Faculty of Science and Technology, Department of Public Health, Cavendish University, Kampala, Uganda; c Faculty of Health Sciences, Department of Public Health Islamic University in Uganda.

## Abstract

**Background:**

Globally, smartphone use among university students is expanding at an exponential rate, and its lingering addiction has now become a global issue, causing some emotional comprehension issues that can lead to significant consequences. Hence, this study aimed to assess the magnitude of smartphone addiction (overuse) and its predictors among health sciences students at selected universities in Kampala, Uganda.

**Methodology:**

An online-based descriptive cross-sectional study design was employed for this study among 308 students of health sciences in Ugandan universities. A three-sectioned, pretested, and validated questionnaire was used to capture data on socio-demographic attributes and smartphone use habits from the respondents. The data were analysed using IBM SPSS version 26. The outcome variable (i.e., smartphone addiction) was transformed into a weighted aggregate score prior to dichotomisation. Analysis of variance, chi-square test of independence, and binary logistic regression analysis were employed for the study hypotheses, and the significance level was set at *P* ≤.05.

**Results:**

The prevalence of smartphone addiction was found to be 53.9%. Female respondents were predominant, 179 (58.1%), and relatively three-quarters of the respondents, 237 (76.9%), were unmarried. The smartphone addiction score among the respondents was 16.13 (95% confidence interval [CI], 15.49 to 16.78) on a maximum reference scale of 30. At the multivariable model, daily time spent using a smartphone (AOR 0.40; 95% CI, 0.23 to 0.69) and the onset of smartphone use (AOR 0.55, 95% CI, 0.31 to 0.97) were identified as the significant independent predictors of smartphone addiction.

**Conclusion:**

This study reported a high prevalence of smartphone addiction among the sampled health sciences students in Ugandan universities. The most significant predictors of smartphone addiction include the number of hours spent on a smartphone daily and the onset of smartphone use. Given the negative health outcomes that this problem may evoke, this study calls for targeted health education intervention to enhance self-control skills, and to effectively tackle smartphone addiction among university students in Uganda.

## BACKGROUND

A smartphone is a mobile phone with more advanced capabilities and computer power than other basic phones. Smartphones have become more affordable and user-friendly due to technological breakthroughs, opening the road for widespread use. This has resulted in an exponential surge in smartphone use around the world. It is estimated that there were more than 2.5 billion smartphone users worldwide in 2018 and this figure is anticipated to rise to 7.516 billion by 2026.^2^

Despite its benefits, smartphones can lead to “excessive” or “compulsive” use, a condition referred to as “smartphone addiction.” ^3^ Smartphone addiction is a global issue^4^ that has been described using several terminologies, including “smartphone overuse,” “mobile phone addiction,” “problematic mobile phone use”^5^, “addiction proneness”^6^, and “excessive use of smartphones”^7^. Recently, smartphone addiction has been characterised in an empirical study to be a behaviour addiction.^8^ Specifically, smartphone use among university students globally is expanding at an exponential rate.^9^ Young people aged between 18 and 34 years are far more likely to own a smartphone.^10^ According to a global mobile consumer survey performed among over 51,000 participants from 32 countries, 93% of participants aged 18 to 24 years old had the highest smartphone ownership and spent the most time on a smartphone.^10^

Furthermore, experts have noted that, in comparison to their older counterparts, adolescent purchasers are becoming more educated about mobile phone capabilities and features of mobile phones.^11^ Numerous surveys show that young people purchase electronic gadgets earlier than other demographic groups.^12^ While immoderate attachment to a smartphone causes emotional comprehension issues that can lead to significant consequences, adolescent smartphone addiction predisposes users to a variety of issues. Excessive smartphone use, for example, endangers physical health by disrupting sleep, reducing vision, creating pain in the cervical spine, wrists, and shoulders, and causing secondary problems due to a lack of exercise.^13^ Undoubtedly, smartphone addiction continues to be a major, lingering public health concern among users.^14,15^ Excessive smartphone use promotes addiction not only by negatively impacting students’ academic performance specifically but also in other forms of daily activities among other users, including their physical and mental health and social relationships, and can result in withdrawal tendency among some users.^15^

Empirically, it is evident that the lingering high prevalence of smartphone addiction among medical students may impair students’ behaviour, create learning burnout, and ultimately, hamper academic performance and graduation. ^9^ As a result, medical students’ smartphone addiction affects not only their physical and mental health but also the doctor-patient relationship and the quality of medical care they will be providing in the future.

The severity of the problems caused by excessive phone use among health sciences students and their academic performance is yet to be explored or receive significant attention across literature in Uganda. However, as science continues to link numerous health issues to smartphone usage, it is critical to be proactive in dealing with any future harmful implications of smartphone use, especially among the students that will then be the nation’s health workforce in the future. Hence, the purpose of this study was to assess the magnitude of smartphone addiction/overuse and its predictors among Health Sciences University students in Uganda, in order to provide pertinent information that can be leveraged to tackle the unintended consequences of smartphone addiction among this vulnerable population group. We hypothesised that smartphone addiction scores will vary across the socio-demographic attributes of the respondents and that certain socio-demographic characteristics will significantly predict smartphone addiction among the study participants.

## METHODS

Online questionnaires were sent to health sciences students at multiple universities offering health science courses in Uganda using the WhatsApp platform and emails where applicable.

### Study Design

An online-based descriptive cross-sectional study design was employed for this study with the administration of pretested structured questionnaires adapted from the Smartphone Addiction Scale Questionnaire (SAS).

### Study Population

The study population consisted of health science students from selected universities in Uganda. These universities included the Islamic University in Uganda (IUIU), Kampala International University (KIU), and Victoria University.

### Study Duration

The online survey took place within 8 weeks (August– September, 2022).

### Sample Size Determination

A total of 308 students participated in the online survey by completing the questionnaire.

### Sampling Technique

The university students volunteered to participate in the study after receiving an electronic invitation, and they were recruited for this online survey employing a snowball sampling approach. The researchers chose a list of WhatsApp groups and student emails comprising health sciences students from Ugandan universities. The link to the online questionnaire was shared via emails and in WhatsApp groups, and participants were encouraged to distribute it to other sub-WhatsApp groups made up of health science in the selected universities in Uganda, thereby making the approach respondent-driven.

### Data Collection

A three-sectioned, pretested, and validated questionnaire was used to capture data from the respondents online. Google Docs was used to compile (a) socio-demographic data, (b) mobile phone use habits, and (c) the English version of the Smartphone Addiction Scale-Short Version (SAS-SV).^16^ The original SAS-SV has ten questions with six answer choices, scored on a Likert scale of four responses: strongly disagree = 0, disagree = 1, agree = 2, and strongly agree = 3 with no single reverse coding. Upon the assessment of smartphone addiction score by computation of the 10 items measuring smartphone addiction into an aggregate weighted score of a 30-point rating scale.

### Ethical Considerations

The Research and Ethics Committee of the Islamic University of Uganda granted ethical approval for this work. Informed consent was obtained from participants in order to participate and complete the questionnaire by giving a lucid explanation of the study objectives and benefits via the link to the online survey, and agreeing to complete the survey denotes their consent to participate in the study. The participants were assured of confidentiality related to the obtained data. Questionnaires were completed anonymously, with no personal identifiers. Only those who consented to participate in the study completed the online questionnaire.

### Data Analysis

Responses were extracted from the Google Docs data sheet, copied to Microsoft Excel spreadsheets, and analysed with IBM SPSS version 26 statistical software. Counts, frequencies, and proportions were analysed descriptively using statements, tables, and figures for categorical study variables, including socio-demographic attributes and all the constructs of the smartphone addiction scale. Furthermore, the transformation of the 10 items measuring smartphone addiction was conducted by computing the scores for each item to obtain a weighted aggregate score, which was used in assessing the level of smartphone addiction. Summaries of descriptive statistics such as mean, standard deviation, percentage mean score, and 95% CI for the mean were employed to describe the transformed variable. To ascertain whether socio-demographic attributes, including age, gender, marital status, nationality, daily time spent on the phone, and onset of smartphone use, predict the risk of smartphone addiction. Analysis of variance (ANOVA) was conducted for the socio-demographic variables against the transformed smartphone addiction variable, and the mean difference for smartphone addiction scores across the different categories of the demographic attributes was reported.

To assess the predictors of smartphone addiction, the composite (i.e., transformed) variable was further dichotomised as “addicted (yes)” if respondents scored ≥16 on the smartphone addiction scale and “not addicted (no)” if respondents scored <16 in line with a previous empirical study. We employed a chi-square test of independence to evaluate the independent association between all the socio-demographic variables and the dichotomised smartphone addiction variable. The extent of the association between these variables was further assessed using binary logistic regression, and the odds of smartphone addiction across the predictor variables were reported. We kept the level of significance at *P* ≤.05.

## RESULTS

### Demographic Characteristics of the Study Respondents

The study captured data from 308 respondents, and more than half (55.8%) of them were between the ages of 18 and 24 years. Female respondents were predominant (58.1%), and relatively three-quarters of the respondents (76.9%) reported to be single. Further, above half of the total students (55.2%) reported to be in their first year of study, and approximately 2 out of every 3 students (64.0%) reported to be local students. In terms of the number of hours spent using smartphones per day, 67.2% of the respondents spend 5 or more hours daily using smartphones, and the majority of the students (73.4%) started using smartphones 5 or more years ago. Upon the assessment of the score for addiction among the respondents, respondents who were aged between 21 and 31 years (16.8), male (16.3), unmarried respondents (16.4), third-year students (17.4), foreign students (16.5), respondents who spent 5 hours or more daily on smartphones (16.9), and those who started the use of smartphones a year ago or less (20.5) demonstrated the highest scores for smartphone addiction (Table 1).

**Table 1:** Demographic Characteristics of the Study Participants

Variable	Respondents in this study N=308		Mean scores and 95% CI on Smartphone Addiction
	Frequency (n)	Percentage (%)	
Age of respondents in years			
18–24	172	55.8	16.11 (15.22–17.00)
25–31	80	26.0	16.80 (15.57–18.03)
32–38	39	12.7	15.79 (14.08–17.51)
≥39	17	5.5	14.00 (10.78–17.22)
Gender			
Female	179	58.1	16.02 (15.15–16.88)
Male	129	41.9	16.29 (15.31–17.28)
Marital Status			
Single	237	76.9	16.40 (15.67–17.13)
Married	71	23.1	15.25 (13.84–16.67)
Year of study			
First	170	55.2	15.51 (14.67–16.34)
Second	59	19.2	16.85 (15.06–18.63)
Third	21	6.8	17.43 (14.57–20.28)
Fourth	36	11.7	17.28 (15.62–18.93)
Fifth	22	7.1	15.95 (13.56–18.35)
Nationality			
Local Students	197	64.0	15.90 (15.08–16.73)
Foreign Students	111	36.0	16.54 (15.48–17.60)
Daily time spent using Smartphone			
1–2 hours	15	4.9	13.33 (10.06–16.61)
3–4 hours	86	27.9	14.73 (13.70–15.76)
≥5 hours	207	67.2	16.92 (16.10–17.74)
Onset of Smartphone use			
≤1 year	6	1.9	20.50 (14.61–26.39)
2–4 years	76	24.7	14.67 (13.30–16.05)
≥5 years	226	73.4	16.51 (15.78–17.24)

### Findings on Smartphone Addiction

Regarding smartphone addiction among the study participants as presented in Table 2, it can be observed that nearly half of the respondents (46.4%) affirmed that they were missing planned work due to smartphone use. More than one-third of the total respondents (44.2%) agreed that they were having a hard time concentrating in class, while doing assignments, or while walking due to the use of smartphones. Relatively one-third of the respondents (34.1%) agreed that they would not be able to stand not having a smartphone, and 4 out of every 10 students agreed that they were feeling impatient and fretful when they were not holding their smartphone. Additionally, approximately 44% of the study participants affirmed that they were always having their smartphone in mind even when they were not using it; the majority of the respondents (40.9%) affirmed that they would give up using their smartphone when their daily life was greatly affected by it, and roughly half of the participants (48.7%) agreed that they were constantly checking their smartphone so as not to miss conversations with other people on social media platforms. Regarding whether the respondents use their smartphones longer than they intended or not, more than half of them (53.2%) agreed that they were using their smartphones longer than they had intended, and 42%, which constitutes the majority, disagreed that the people around them tell them that they use their smartphones too much (Table 2).

**Table 2:** Findings on Smartphone Addiction among the Respondents

Items	Respondents in this study N= 308							
	Strongly Agree		Agree		Disagree		Srongly Disagree	
	N	%	N	%	N	%	N	%
Missing planned work due to smartphone use	57	18.5	143	46.4	70	22.7	38	12.3
Having hard-time concentrating in class, while doing assignments, or while walking due to smartphone use	52	16.9	136	44.2	88	28.6	32	10.4
Feeling pain in the wrist or at the back of the neck while using smartphone	20	6.5	139	45.1	121	39.3	28	9.1
I won't be able to stand not having a smartphone	72	23.4	105	34.1	91	29.5	40	13.0
Feeling inpatient and fretful when I am not holding my smartphone	47	15.3	124	40.3	103	33.4	34	11.0
Having my smartphone in my mind even when I am not using it	45	14.6	135	43.8	94	30.5	34	11.0
I will never give up using my smartphone even when my daily life is greatly affected by it	31	10.1	85	27.6	126	40.9	66	21.4
Constantly checking my smartphone so as not to miss conversations with other people on WhatsApp, Twitter or Facebook, etc.	57	18.5	150	48.7	67	21.8	34	11.0
Using my smartphone longer than I had intended	74	24.0	164	53.2	49	15.9	21	6.8
The people around me tell me that I use my smartphone too much	48	15.6	80	26.0	129	41.9	51	16.6

**Table 3:** Prevalence and Summaries of Descriptive Statistics Computed for Smartphone Addiction

Variables	Maximum score on rating scale	Mean (±SD)	95% Confidence Interval	Percentage mean score	Prevalence of Smartphone Addiction	
Smartphone Addiction	30	16.13 (5.77)	15.49–16.78	53.8%	Addicted N (%) 166 (53.9)	Addicted N (%) 142 (46.1)

### Prevalence of Smartphone Addiction among the Study Respondents

**Figure 1:**
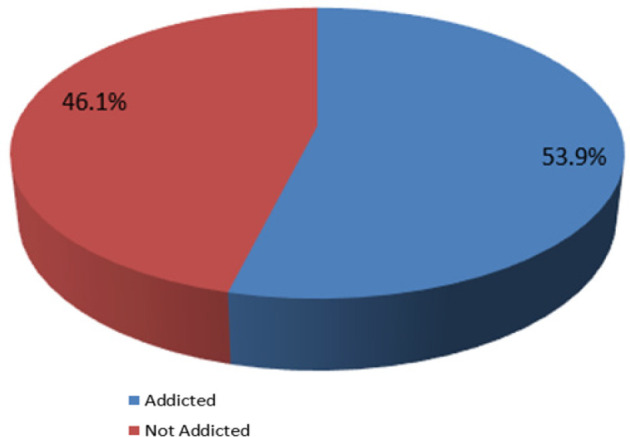
Prevalence of Smartphone Addiction

Upon the assessment of smartphone addiction score by computation of the 10 items measuring smartphone addiction into an aggregate weighted score of a 30-point rating scale, the respondents had a general mean score of 16.13 (95% CI, 15.49 to 16.78), SD of 5.77, and percentage mean score of 53.8% computed for smartphone addiction; the prevalence of addiction among the respondents is observed to be 53.9%, which denotes that more than half of the respondents were addicted to the use of smartphones (Figure 1).

### Factors associated with Smartphone Addiction among the Study Respondents

The findings on the predictors of smartphone addiction among the study participants showed that at the bivariate level, positive insignificant associations exist between the age of the respondents, the nationality of the respondents, and smartphone addiction. However, gender, marital status, and students’ year of study are negatively associated with smartphone addiction, although the associations are not statistically significant. The only two significant predictors of smartphone addiction at the bivariate level are total daily time spent using a smartphone and the onset of smartphone use, where it can be observed that respondents who spend 1–2 hours per day using a smartphone are 67% less likely to be addicted to the use of a smartphone (COR 0.33, 95% CI, 0.11 to 0.99) and the odds of smartphone addiction decrease by 53% among respondents who spend 3–4 hours daily using a smartphone (COR 0.47, 95% CI, 0.28 to 0.78). Furthermore, respondents who started using smartphones 2–4 years ago had a 54% lower likelihood of being addicted to the use of smartphones (COR 0.46, 95% CI, 0.27 to 0.79) compared to those who have been using smartphones for the last 5 years or more. In a multivariable model, daily time spent using a smartphone and the onset of smartphone use remain significantly associated with smartphone addiction. The findings showed that respondents who spend 3 to 4 hours daily using smartphones (AOR 0.40, 95% CI, 0.23 to 0.69) are 60% less likely to be addicted to the use of smartphones, which implies they are 2.5 times more likely to use smartphones wisely compared to their counterparts. Similarly, the odds of smartphone addiction decrease by 45% among students who started using smartphones 2 to 4 years ago (AOR 0.55, 95% CI, 0.31 to 0.97). Table 4 describes the predictors of smartphone addiction among the study participants.

**Table 4:** Factors Associated with Smartphone Addiction among the Study Participants

Variables	Respondents in this study N=308			*P-value*	*AOR (95% CI)*	*p-value*
	Smartphone Addiction		Crude Odds Ratio (95% CI)			
	Yes n (%)	No n (%)				
Age of Participants (in years)						
18–24	87 (52.4)	85 (59.9)	1.46 (0.53–4.02)	.641	0.66 (0.19–2.27)	.508
25–31	50 (30.1)	30 (21.1)	2.38 (0.82–6.92)	.111	1.24 (0.37–4.16)	.724
32–38	22 (13.3)	17 (12.0)	1.85 (0.58–5.87)	.297	1.23 (0.35–4.29)	.750
≥39	7 (4.2)	10 (7.0)	1		1	
Gender						
Female	94 (56.6)	85 (59.9)	0.86 (0.56–1.69)	.929	0.81 (0.49–1.34)	.409
Male	72 (43.4)	57 (40.1)	1		1	
Marital status						
Married	36 (21.7)	35 (24.6)	0.85 (0.50–1.44)	.539	0.68 (0.35–1.35)	.270
Single	130 (78.3)	107 (75.4)	1		1	
Year of Study						
First	85 (51.2)	85 (59.9)	0.69 (0.28–1.71)	.424	0.96 (0.35–2.65)	.943
Second	31 (18.7)	28 (19.7)	0.77 (0.28–2.07)	.599	1.09 (0.37–3.20)	.881
Third	16 (9.6)	5 (3.5)	2.22 (0.59–8.26)	.236	2.99 (0.73–12.26)	.129
Fourth	21 (12.7)	15 (10.6)	0.97 (0.33–2.85)	.955	1.21 (0.38–3.90)	.744
Fifth	13 (7.8)	9 (6.3)	1		1	
Nationality						
Foreign students	65 (39.2)	46 (32.4)	1.34 (0.84–2.15)	.218	1.67 (0.94–2.97)	.082
Local students	101 (60.8)	96 (67.6)	1		1	
Total Daily Time Spent using Smartphone (in hours)						
1–2	5 (3.0)	10 (7.0)	0.33 (0.11–0.99)	.049*	0.37 (0.11–1.19)	.094
3–4	36 (21.7)	50 (35.2)	0.47 (0.28–0.78)	.004*	0.40 (0.23–0.69)	.001*
≥5	125 (75.3)	82 (57.7)	1		1	
Onset of Smartphone use						
≤1 year	4 (2.4)	2 (1.4)	1.42 (0.26–7.94)	.687	2.75 (0.43–17.78)	.288
2–4 years	30 (18.1)	46 (32.4)	0.46 (0.27–0.79)	.005*	0.55 (0.31–0.97)	.040*
≥5 years	132 (58.4)	94 (66.2)	1		1	

## DISCUSSION

This present study captured pertinent data on the burden and predictors of smartphone addiction among health sciences students across different universities in Uganda. The report from this current study revealed that out of 308 respondents, the overall smartphone addiction prevalence was relatively above average at 166 (53.9%). This is slightly higher compared to previous similar studies conducted in South India, which reported 46.1% and 36.8% prevalence of addiction.^17,18^, respectively. Similar lower rates of approximately 30% have been reported in the studies conducted in Asia.^16,19^ while using the same smartphone addiction scale-short version. However, the prevalence of smartphone addiction in our study was less than that observed in a similar study on smartphone addiction among medical students in the Andaman and Nicobar Islands by Sethuraman and others^20^, which reported an 85% smartphone addiction rate among their respondents. One of the reasons for this high prevalence in our study could be the need for students to access their study materials on their smartphones, especially in adjusting to the blended system of learning that was introduced in many countries immediately following the aftermath of the unprecedented COVID-19 pandemic.

Demographically, it is imperative to consider the gender differences observed in the level of smartphone addiction; male students were slightly more predisposed to the risk of smartphone addiction by displaying the highest score for addiction compared to females in this study, although it was not statistically significant. In corroboration with this present study, findings from various empirical studies revealed varied results on female gender being more likely to be addicted to smartphone use compared to male students, yet the high prevalence observed among their female respondents is not statistically significant^21,22^. This is an indication that smartphone addiction is not gender-specific. Therefore, interventions that will be tailored to address this prevailing issue in this setting should be an inclusive and concerted approach that leaves no gender groups untouched.

Furthermore, upon the assessment of the score for addiction among the respondents, those aged between 21 and 31 years had a higher tendency to be smartphone addicts compared to respondents of other age groups. However, there was no significant association between the age of respondents and smartphone addiction. This finding is closely in line with the findings from the study conducted by Poushter,^10,19^ where it was demonstrated that young adults aged 18 to 34 years were much more likely to be addicted smartphone users.

Looking at our observed addiction score in this study, it shows that unmarried respondents are more addicted to smartphones than the married respondents, although our study did not reveal any association between marital status and smartphone addiction statistically. The reason for this could be that the married respondents have marital commitments that will preoccupy them most of the time and avail them limited time to use and spend on the phone compared to the unmarried respondents, who might be less preoccupied with much domestic responsibilities.

Additionally, our study revealed a statistically significant relationship between the daily hours spent on smartphones and smartphone addiction in agreement with the findings conducted in Switzerland, Turkey, and Korea.^23,24,11^ In terms of the number of hours spent using a smartphone per day, 67.2% of the respondents spend 5 hours or more daily using a smartphone. This implies that respondents who spend more than 3 hours per day using smartphones are 2.5 times more likely to be addicted to the use of smartphones than respondents who spend less than 2 hours per day using smartphones. This possibly indicates that respondents were spending considerable time on their smartphones due to a lot of social media and educative applications they need to access daily. However, this overuse or overdependence can have a negative impact on the respondents’ academic performance; undoubtedly, as medical students, they need more time to study.

Again, there was a significant relationship between the number of years spent using smartphones and smartphone addiction among the respondents, where respondents who started using smartphones less than 5 years ago (i.e., 2–4 years ago) were less likely to be addicted to the use of smartphones compared to those who have been using smartphones for 5 years or more. This implies that phone use habit formation has shaped the dynamics of addiction among the respondents in this study. Consequently, targeted health education interventions that will be geared towards enhancing the self-control skills of the students should be embraced in subsequent public health campaigns in order to reduce the lingering burden of smartphone addiction in this setting and promote healthy smartphone use behaviour among the vulnerable student population.

## CONCLUSION

This study reported a high prevalence of smartphone addiction among the sampled health sciences students in Ugandan universities. The most significant predictors of smartphone addiction include daily hours spent on a smartphone and the onset of smartphone use. Given the negative health outcomes that this problem may evoke, targeted health education interventions that will be geared towards enhancing the self-control skills of the students should be embraced in subsequent public health campaigns in order to reduce the lingering burden of smartphone addiction in this setting and promote healthy smartphone use behaviour among the vulnerable student population.

### Limitations

Due to the sampling strategy (snowball sampling) employed in this present study, we are aware of the difficulties encountered by the initial responders from the key WhatsApp groups to whom our survey items were distributed. However, we were unable to determine the ultimate breadth of the online questionnaire’s distribution to students across Kampala’s health science universities. Without a doubt, this study highlights that there is a need to conduct more robust longitudinal studies across health science students in Uganda as a diagnostic approach to having a deepened understanding of the probable reasons surrounding the overuse of the smartphone devices among the students.
